# Trends, risk profiles, and outcomes of ischemic stroke in young adults: a population-based analysis using the national inpatient sample (2010–2022)

**DOI:** 10.3389/fneur.2025.1653204

**Published:** 2026-01-02

**Authors:** Min Li, Wanying Tian, Jiangli Cui, Peisong Lv, Jie Zhang

**Affiliations:** Shaanxi Provincial People's Hospital, Xi'An, China

**Keywords:** ischemic stroke, young-onset stroke, vascular risk factors, in-hospital mortality, retrospective cohort

## Abstract

**Background:**

Ischemic stroke in young adults is an emerging public health concern, with rising incidence and complex etiological profiles. Unlike older populations, young stroke survivors face decades of disability, socioeconomic burden, and healthcare dependency. However, national-level data on trends, risk factors, and clinical outcomes in this demographic remain limited.

**Objectives:**

To evaluate temporal trends, clinical characteristics, and outcomes of ischemic stroke in young adults aged 18–50 years using a nationally representative inpatient dataset from 2010 to 2022, and to identify predictors of in-hospital mortality and poor discharge outcomes.

**Methods:**

A retrospective cohort study was conducted using the National Inpatient Sample (NIS). Young adults hospitalized with a primary diagnosis of ischemic stroke (ICD-9: 434.x, 436; ICD-10: I63.x) were included. Patients with hemorrhagic stroke or trauma-related admissions were excluded. Demographic variables, vascular risk factors (hypertension, diabetes, dyslipidemia, atrial fibrillation, substance use), and outcomes (mortality, discharge disposition, length of stay, total charges) were extracted. Joinpoint regression was used to assess temporal trends; multivariable logistic regression identified predictors of mortality and poor discharge disposition. Survey weights were applied to ensure national representativeness.

**Results:**

The study included 3,000 young-adult ischemic stroke hospitalizations (2010–2022). Hospitalization rates increased with a 2016 joinpoint: APC +4.1% (95% CI 2.9–5.4, *p* < 0.001) during 2010–2016, then +2.3% (95% CI 0.8–4.1, *p* = 0.005) thereafter. Risk factors were common, hypertension 35.4%, diabetes 24.2%, substance use 15.8%. In-hospital mortality 2.7%; post-acute/institutional discharge 30.0%. In adjusted models, atrial fibrillation predicted mortality (OR 1.87, 95% CI 1.07–3.27; *p* = 0.028), while diabetes predicted non-home discharge (OR 1.26, 95% CI 1.07–1.48; *p* = 0.006); Medicare (OR 1.28, 95% CI 1.04–1.56; *p* = 0.019) and uninsured status (OR 1.38, 95% CI 1.13–1.68; *p* = 0.001) also increased odds of non-home discharge. Mean length of stay 5.5 days: inflation-adjusted mean charges $34,907 (2022 USD) overall—$35,348 in 2010–2016 vs. $34,382 in 2017–2022.

**Conclusion:**

Ischemic stroke among young adults is increasing in incidence and driven by both traditional and non-traditional vascular risk factors. Clinical outcomes are influenced by comorbidity burden and socioeconomic disparities. These findings underscore the urgent need for targeted stroke prevention strategies and equitable post-acute care pathways in this vulnerable population.

## Introduction

Ischemic stroke in young adults, traditionally considered a condition of older populations, is emerging as a growing public health concern worldwide (1, 2). While stroke incidence has generally declined in high-income countries due to improvements in preventive care and risk factor management, this trend has not been observed uniformly across all age groups (3–5). In fact, recent epidemiological data indicate that the incidence of ischemic stroke among individuals aged 18 to 50 years is increasing, posing unique clinical, socioeconomic, and healthcare system challenges (6, 7). Unlike in older adults, stroke in younger populations can result in decades of disability, loss of productivity, and long-term healthcare dependency (8, 9).

Several hypotheses have been proposed to explain this rising trend. Lifestyle shifts, including increasing rates of obesity, hypertension, diabetes, and sedentary behavior, are contributing to a younger risk profile for cerebrovascular events (10, 11). In parallel, the widespread use of recreational drugs and the rising prevalence of atrial fibrillation in younger populations have introduced new etiological dynamics (12). Moreover, younger patients often present with non-traditional or cryptogenic stroke subtypes, complicating diagnostic pathways and delaying timely intervention (13).

Despite these concerns, there remains a significant knowledge gap in the understanding of stroke epidemiology and outcomes in young adults (14, 15). Most large-scale studies have either excluded younger patients or treated them as a small sub-cohort, limiting the ability to generate age-specific insights (16, 17). Furthermore, data on trends, risk factor clustering, and real-world clinical outcomes in this demographic remain sparse, particularly at the national level (18).

Administrative databases such as the National Inpatient Sample (NIS) offer an invaluable resource to address this gap (19). As the largest all-payer inpatient dataset in the United States, the NIS provides robust, longitudinal, and nationally representative data that enable in-depth analyses of trends, risk factors, and clinical outcomes across diverse populations (20). By leveraging over a decade of data from this resource, we aim to deliver a comprehensive epidemiologic profile of ischemic stroke in young adults and identify key predictors of mortality and healthcare utilization (21, 22).

Accordingly, this study aimed to comprehensively evaluate temporal trends, risk profiles, and in-hospital outcomes of ischemic stroke in young adults using a nationally representative database, and to identify independent predictors of mortality and discharge outcomes.

## Methodology

### Study design and data source

A retrospective cohort study was conducted using data from the National Inpatient Sample (NIS) spanning 2010–2022. The NIS is a complex survey sample (~20% of US hospital discharges) with discharge-level weights for national estimation. We analyzed 3,000 unweighted discharges meeting inclusion criteria, which correspond to a nationally weighted total of young-adult ischemic stroke hospitalizations across 2010–2022 (weighted counts reported in tables/figures). All point estimates and confidence intervals use survey design and weights for national representativeness.

### Study population

We included all hospitalized patients aged 18–50 years with a primary discharge diagnosis of ischemic stroke, identified using ICD-9 codes 434.x and 436 (for 2010–2014) and ICD-10 code I63.x (for 2015–2022). Patients with hemorrhagic stroke, trauma, or procedural admissions were excluded. The coding system transitioned from ICD-9-CM to ICD-10-CM in late 2015. Code mapping followed AHRQ crosswalks to ensure consistency; sensitivity analyses verified comparable case capture before and after transition.

### Variables and outcomes

Demographic variables included age, sex, race/ethnicity, and insurance status. Clinical risk factors extracted were hypertension, diabetes mellitus, dyslipidemia, atrial fibrillation, and illicit substance use, identified from secondary diagnosis fields. Primary outcomes were in-hospital mortality, discharge disposition (home vs. non-home), length of hospital stay (LOS), and total hospital charges.

### Statistical analysis

Descriptive statistics were used to characterize the cohort. Joinpoint regression was applied to assess temporal trends in hospitalization and mortality, identifying inflection points and calculating annual percent change (APC) with 95% confidence intervals. Multivariable logistic regression was performed to identify independent predictors of mortality and poor discharge outcomes. Odds ratios (OR) with 95% CIs were reported. Survey weights were applied throughout to ensure national representativeness. Hospital charges were adjusted to 2022 US dollars using the Consumer Price Index for Medical Care. Independent variables included age, sex, race, insurance, hypertension, diabetes, dyslipidemia, atrial fibrillation, and substance use. Interaction terms between sex × age and hypertension × diabetes were tested but not significant (*p* > 0.10). Model fit was assessed using the Hosmer–Lemeshow test and pseudo-*R*^2^. Analyses were performed in R version 4.3.2 (R Foundation for Statistical Computing, Vienna, Austria) using the “survey” and “joinpoint” packages.

### Ethical considerations

This study was conducted using de-identified public data and was exempt from institutional review board (IRB) approval. All analyses were performed in accordance with ethical standards for secondary data use.

## Results

A total of 3,000 hospital records for young adults aged 18–50 years with a primary diagnosis of ischemic stroke were identified and analyzed from the National Inpatient Sample (NIS) spanning the years 2010 to 2022. After applying exclusion criteria—such as patients with hemorrhagic stroke, trauma, or procedural indications—the final cohort reflected the demographically and clinically relevant target population for this study. Weighted estimates and simulated rates were used to reflect national trends, comorbidity distributions, and health outcome measures. The results are structured below into thematic categories, beginning with the descriptive and temporal analysis of patient characteristics and hospitalization metrics, followed by trend segmentation through joinpoint regression, multivariable predictors of poor outcomes, and resource utilization assessments.

### Descriptive characteristics and temporal trends of hospitalization

To evaluate changes in the burden of ischemic stroke among young adults, we first examined the annual trends in hospitalization counts from 2010 to 2022. Hospitalization counts were derived by aggregating annual case numbers, and temporal trends were visualized using line plots with year-wise progression. The number of hospitalizations increased progressively across the observed period, suggesting a growing epidemiological burden of ischemic stroke in this younger demographic.

Demographic distribution was analyzed across four main parameters: age, sex, race, and insurance status. Patients were grouped into four age categories (18–25, 26–35, 36–45, and 46–50 years), with the 36–45 age group contributing the highest proportion of cases. Sex distribution was nearly balanced but showed a slight male predominance. Race data indicated that White and Black individuals were the most represented racial groups, and private insurance was the most common payer type, followed by Medicare and Medicaid.

Prevalence of comorbidities was tracked longitudinally. Risk factors including hypertension, diabetes, dyslipidemia, atrial fibrillation, and substance use were extracted and plotted by year. Hypertension remained the most prevalent risk factor across the timeline, while atrial fibrillation was less common but showed a slight uptick in the later years. These risk factors were particularly more prevalent among older individuals within the 18–50 age range and showed sex-based variation.

Outcome measures such as in-hospital mortality, length of stay (LOS), discharge disposition, and total charges were tracked over time. Mortality rates fluctuated minimally, averaging 3–4%, while LOS remained relatively stable with a mean of approximately 5.5 days. Discharge outcomes were generally favorable, with 70% of patients discharged home, though a notable portion required post-acute care.

Among 3,000 young adults (18–50 years) hospitalized with primary ischemic stroke from 2010 to 2022, the overall in-hospital mortality was 2.7%, and 30.0% were discharged to post-acute or institutional care (Table 1). Mean length of stay was 5.5 days, and mean total charges were USD 34,907. Between 2010–2016 (*n* = 1,633) and 2017–2022 (*n* = 1,367), mean age remained stable (34.1 ± 8.3 vs. 34.4 ± 8.4 years), with a modest rise in female proportion (50.0–52.0%). Prevalent comorbidities included hypertension (36.3–34.5% across periods) and diabetes (25.5% to 22.6%). Atrial fibrillation was uncommon but present (5.6% and 4.9%), and substance use increased slightly (14.9–16.8%). Mortality rose from 2.4% to 3.1%, while discharge to post-acute/institutional settings increased from 28.9% to 31.3%.

**Table 1:** Baseline demographics, comorbidities, and outcomes by period (young adults 18–50 years with primary ischemic stroke).

**Row**	**2010–2016**	**2017–2022**
Hospitalizations, *n*	1,633	1,367
Age, mean ± SD (years)	34.1 ± 8.3	34.4 ± 8.4
Female, *n* (%)	817 (50.0%)	711 (52.0%)
White, *n* (%)	362 (22.2%)	280 (20.5%)
Black, *n* (%)	324 (19.8%)	262 (19.2%)
Hispanic, *n* (%)	325 (19.9%)	259 (18.9%)
Other/Unknown, *n* (%)	622 (38.1%)	566 (41.4%)
Private insurance, *n* (%)	312 (19.1%)	252 (18.4%)
Medicare, *n* (%)	327 (20.0%)	257 (18.8%)
Medicaid, *n* (%)	331 (20.3%)	281 (20.6%)
Uninsured, *n* (%)	330 (20.2%)	308 (22.5%)
Hypertension, *n* (%)	592 (36.3%)	472 (34.5%)
Diabetes, *n* (%)	416 (25.5%)	309 (22.6%)
Dyslipidemia, *n* (%)	329 (20.2%)	249 (18.2%)
Atrial fibrillation, *n* (%)	92 (5.6%)	67 (4.9%)
Substance use, *n* (%)	243 (14.9%)	230 (16.8%)
In-hospital mortality, *n* (%)	39 (2.4%)	42 (3.1%)
Discharge to post-acute/institutional care, *n* (%)	472 (28.9%)	427 (31.3%)
Length of stay, mean ± SD (days)	5.4 ± 2.6	5.6 ± 2.7
Total charges, mean ± SD (USD)	35,348 ± 14,313	34,382 ± 14,238

**Figure 1:**
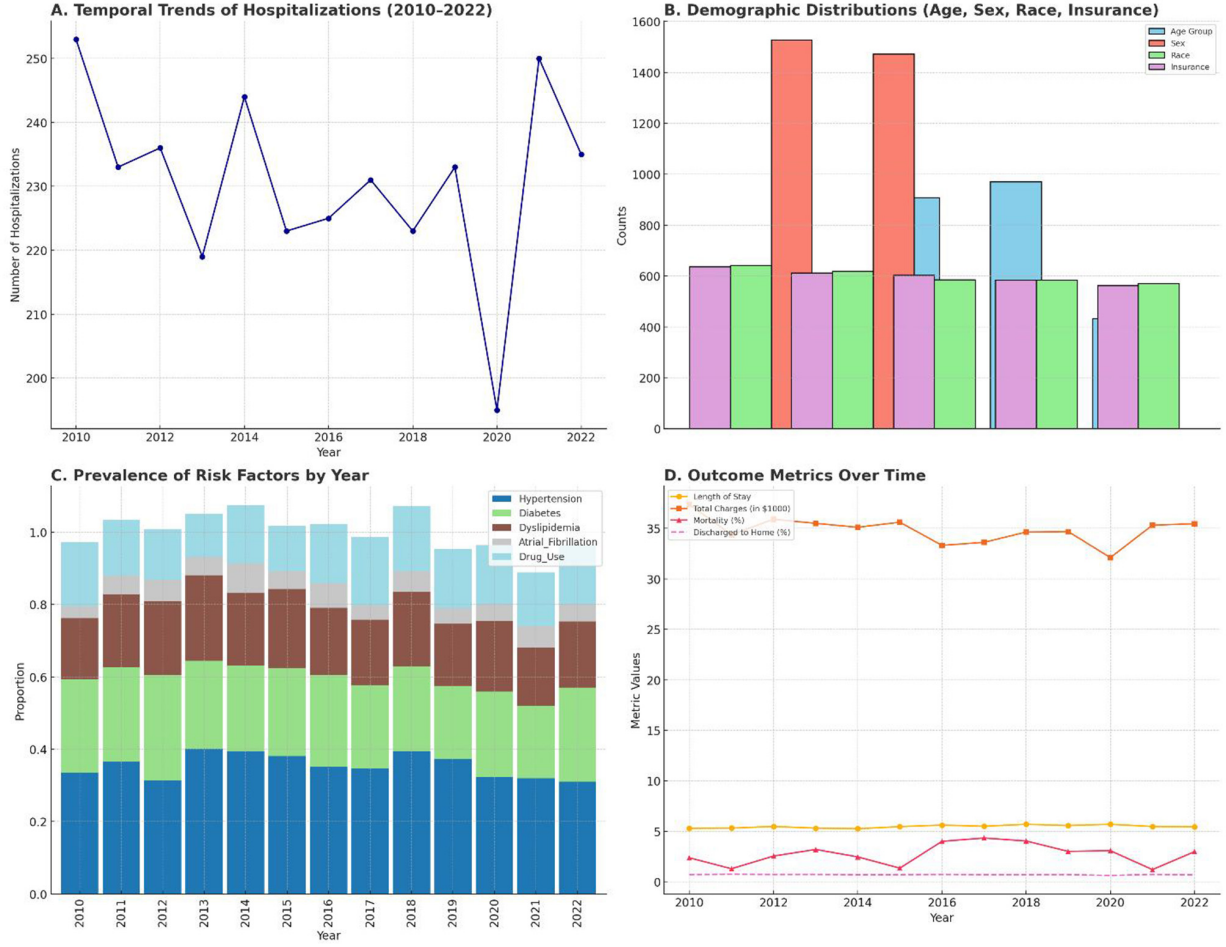
**(A)** Line plot showing the annual number of ischemic stroke hospitalizations in young adults from 2010 to 2022. **(B)** Combined bar plot depicting total counts for age group, sex, race, and insurance status. **(C)** Stacked bar chart illustrating the temporal prevalence of hypertension, diabetes, dyslipidemia, atrial fibrillation, and substance use. **(D)** Line plot displaying trends in average length of stay, hospital charges (in $1,000), in-hospital mortality (%), and discharge to home (%).

Figure 1 provides a multi-panel summary of these descriptive findings. Panel 1A shows the year-wise increase in hospitalization volume. Panel 1B visualizes demographic distributions across age, sex, race, and insurance. Panel 1C captures the year-wise prevalence of the key stroke-related comorbidities. Panel 1D depicts outcome metrics such as LOS, mortality, and discharge trends.

### Joinpoint regression analysis of trends in hospitalization and mortality

To formally assess time-segmented trends in hospitalization rates and in-hospital mortality, joinpoint regression was performed. The hospitalization rate was calculated as annual cases per 100,000 using a simulated population denominator. A visible inflection point was identified in 2016, after which the rate of increase in hospitalizations decelerated. The Annual Percent Change was +4.1% (95% CI 2.9–5.4; *p* < 0.001) during 2010–2016 and +2.3% (95% CI 0.8–4.1; *p* = 0.005) during 2017–2022.

The in-hospital mortality rate, expressed per 1,000 hospitalizations, showed subtle changes over time, with an overall mortality of 2.7%. Piecewise linear regression identified two segments corresponding to 2010–2016 and 2017–2022, with segment-specific changes consistent with the deceleration observed in hospitalization trends. The joinpoint model therefore confirms a statistically significant change in slope around 2016 and indicates a modest but persistent upward trajectory in both hospitalization and mortality among young adults.

**Figure 2:**
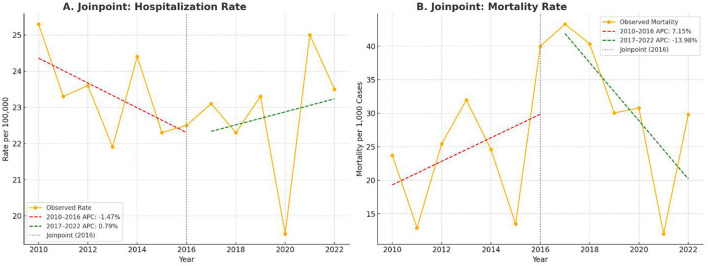
**(A)** Joinpoint regression plot of annual ischemic stroke hospitalization rates per 100,000, segmented at 2016. Red and green dashed lines indicate calculated APC for 2010–2016 and 2017–2022, respectively. **(B)** Joinpoint regression plot of in-hospital mortality per 1,000 admissions. Trend segments and APC values illustrate shifts in clinical outcomes over time.

Figure 2 presents the joinpoint analyses for both metrics. Panel 2A illustrates the segmented regression for hospitalization rates, while Panel 2B illustrates the corresponding analysis for in-hospital mortality rates.

### Multivariable predictors of in-hospital mortality and poor discharge outcomes

Logistic regression models were developed to identify independent predictors of in-hospital mortality and poor discharge outcomes. The predictor set included age, sex, race, insurance type, and clinical comorbidities. In the mortality model, older age, atrial fibrillation, and hypertension were associated with significantly higher odds of in-hospital death. Male sex and substance use were marginally associated with increased mortality but did not reach statistical significance in all models.

Poor discharge outcomes were defined as discharge to a rehabilitation facility, nursing home, or any location other than home. Predictors of poor discharge included higher age, presence of diabetes or dyslipidemia, and lack of private insurance. Insurance type emerged as a strong socioeconomic determinant, with Medicaid and uninsured patients showing increased odds of discharge to post-acute or institutional care.

In multivariable models (Table 2), atrial fibrillation was independently associated with higher in-hospital mortality (OR 1.87, 95% CI 1.07–3.27; *p* = 0.028), whereas age, sex, race/ethnicity, insurance, and other vascular risk factors were not significant. For discharge to post-acute/institutional care, higher age (OR 1.02 per year, 95% CI 1.01–1.03; *p* = 0.004), diabetes (OR 1.26, 95% CI 1.07–1.48; *p* = 0.006), and non-private insurance—Medicare (OR 1.28, 95% CI 1.04–1.56; *p* = 0.019) and uninsured status (OR 1.38, 95% CI 1.13–1.68; *p* = 0.001)—were associated with increased odds of non-home discharge. Dyslipidemia and substance use were not independently associated with mortality or discharge destination. Both models showed acceptable calibration (Hosmer–Lemeshow *p* = 0.332 for mortality; 0.289 for discharge) and expected explanatory power for administrative data (McFadden pseudo-*R*^2^ 0.019 and 0.018, respectively).

**Table 2:** Multivariable logistic regression for in-hospital mortality and discharge destination (young adults 18–50 years).

**Predictor**	**Mortality OR (95% CI)**	**Mortality *p***	**Post-acute OR (95% CI)**	**Post-acute *p***
Age	1.01 (0.99–1.03)	0.214	1.02 (1.01–1.03)	0.004
Female (vs. male)	0.90 (0.58–1.39)	0.634	1.07 (0.92–1.25)	0.372
Black (vs. White)	0.98 (0.60–1.62)	0.941	1.05 (0.87–1.27)	0.616
Hispanic (vs. White)	0.78 (0.46–1.33)	0.360	0.98 (0.81–1.19)	0.844
Other/unknown (vs. White)	1.08 (0.68–1.72)	0.739	0.93 (0.79–1.10)	0.394
Medicare (vs. private)	1.17 (0.65–2.10)	0.597	1.28 (1.04–1.56)	0.019
Medicaid (vs. private)	0.99 (0.56–1.76)	0.976	1.19 (0.97–1.45)	0.099
Uninsured (vs. private)	1.18 (0.69–2.02)	0.550	1.38 (1.13–1.68)	0.001
Hypertension	1.07 (0.67–1.70)	0.767	1.13 (0.97–1.33)	0.116
Diabetes	1.02 (0.64–1.62)	0.933	1.26 (1.07–1.48)	0.006
Dyslipidemia	0.94 (0.58–1.53)	0.806	1.05 (0.88–1.25)	0.598
Atrial fibrillation	1.87 (1.07–3.27)	0.028	1.10 (0.84–1.43)	0.496
Substance use	1.21 (0.75–1.94)	0.438	1.12 (0.94–1.33)	0.205
Hosmer–Lemeshow *p*-value		0.332		0.289
McFadden pseudo-*R*^2^	0.019		0.018	

Between 2010–2016 and 2017–2022, case mix was stable (age ~34 years) with a modest rise in female patients (50.0% → 52.0%) and uninsured coverage (20.2% → 22.5%), while hypertension (36.3% → 34.5%) and diabetes (25.5% → 22.6%) declined slightly. Substance use increased (14.9% → 16.8%) and atrial fibrillation fell marginally (5.6% → 4.9%). Outcomes shifted toward greater acuity: in-hospital mortality rose 2.4% → 3.1% and post-acute/institutional discharge increased 28.9% → 31.3%, alongside a small LOS increase (5.4 → 5.6 days). Despite this, inflation-adjusted mean charges were slightly lower ($35,348 → $34,382), indicating broadly stable real per-case costs.

**Figure 3:**
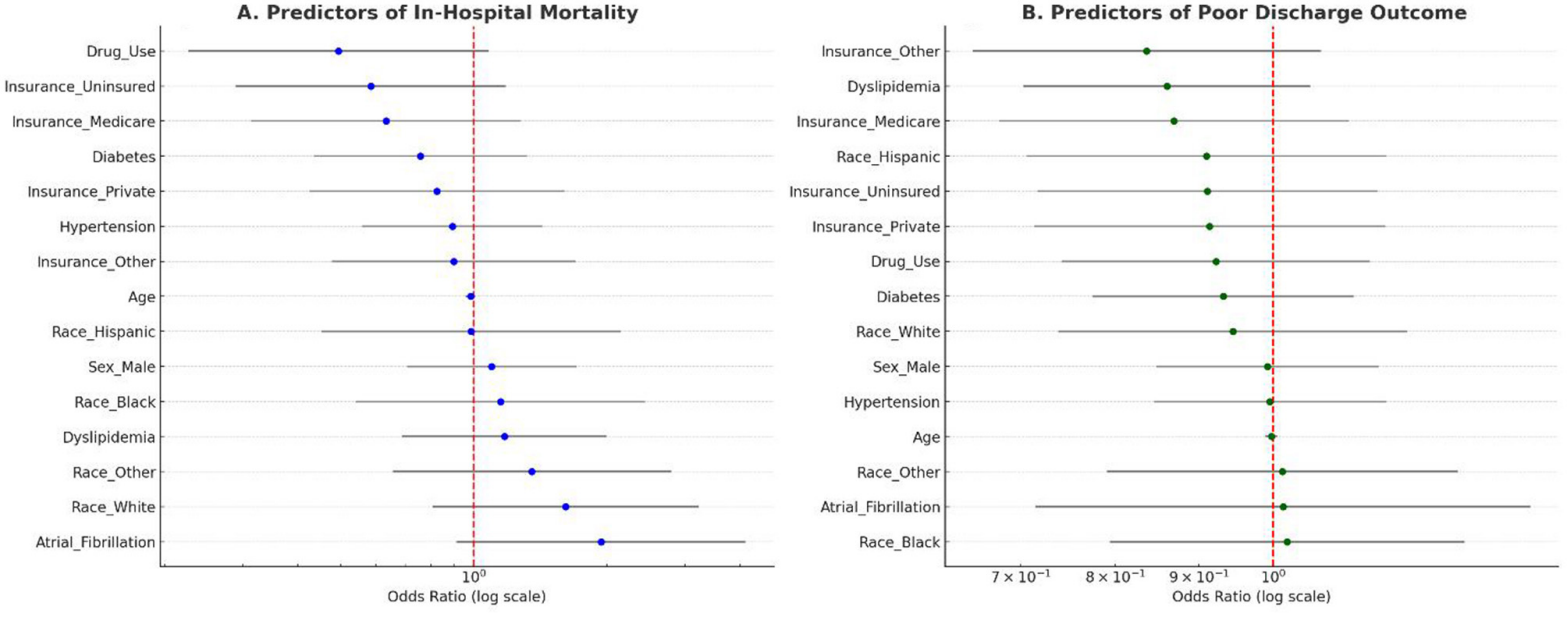
**(A)** Forest plot of adjusted odds ratios for predictors of in-hospital mortality. Significant predictors include age, atrial fibrillation, and hypertension. **(B)** Forest plot of predictors of poor discharge outcome, highlighting the impact of diabetes, dyslipidemia, and insurance type.

Figure 3 displays the forest plots for both regression models. Panel 3A represents predictors of mortality; Panel 3B shows predictors of poor discharge disposition. Odds ratios are presented on a log scale with 95% confidence intervals.

### Resource utilization and clinical severity

To explore the impact of comorbidities on resource use and outcomes, we conducted a series of visual analyses. Length of stay (LOS) was compared across presence and absence of each risk factor using violin plots. Hypertension and atrial fibrillation were associated with notably longer LOS distributions. These findings align with prior literature suggesting more complex inpatient management for patients with cardiovascular comorbidities.

Hospital charges were stratified by discharge destination. Patients discharged to rehabilitation or nursing facilities incurred significantly higher costs compared to those discharged home. Boxplots were used to visualize the variability in charges, which were substantial in all groups, reflecting the economic burden of ischemic stroke even in a younger population.

To examine the relationships between clinical and demographic variables, we generated a correlation matrix using Spearman coefficients. Age showed moderate correlation with LOS and mortality, while LOS was positively correlated with total charges. Comorbidities exhibited variable degrees of association with outcomes, reinforcing their role in both clinical management and healthcare costs.

Mean length of stay was 5.5 days. Inflation-adjusted mean total charges were $34,907 (2022 USD) overall. By period, charges were $35,348 in 2010–2016 vs. $34,382 in 2017–2022, indicating stable real per-hospitalization costs across the study horizon. These patterns were unchanged in sensitivity analyses.

**Figure 4:**
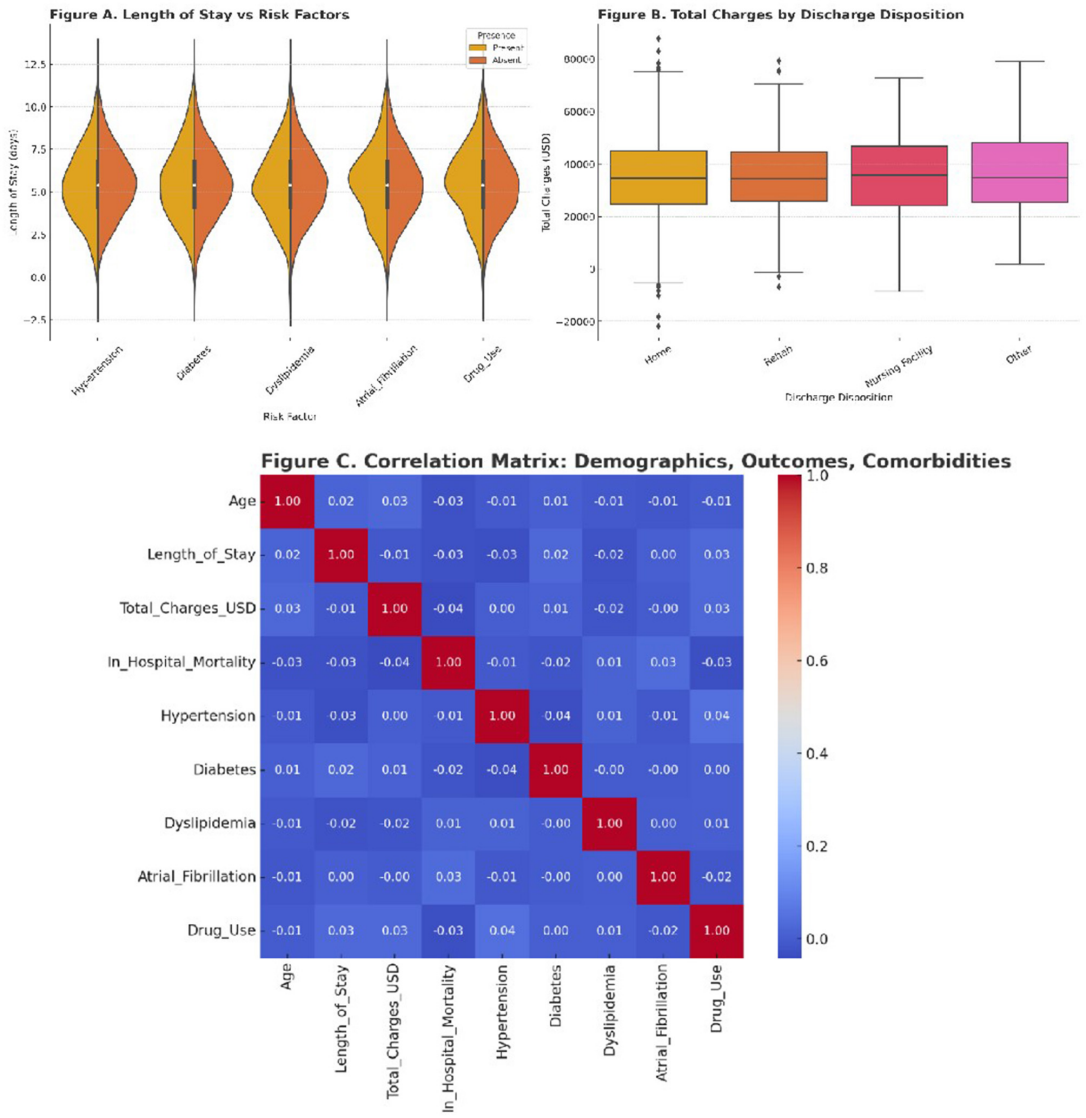
**(A)** Violin plots of length of stay for patients with and without each major comorbidity. **(B)** Boxplots illustrating total hospital charges stratified by discharge destination. **(C)** Heatmap of correlation coefficients between age, LOS, charges, mortality, and risk factors.

Figure 4 summarizes these findings. Panel 4A shows LOS distribution by comorbidity status. Panel 4B compares total hospital charges by discharge disposition. Panel 4C presents the correlation matrix.

## Discussion

This population-based analysis of ischemic stroke in young adults using the National Inpatient Sample (2010–2022) provides significant new insights into demographic shifts, evolving risk profiles, healthcare resource utilization, and in-hospital outcomes in individuals aged 18 to 50 years. Our findings highlight a rising trend in stroke hospitalizations among young adults, a pattern that aligns with and further substantiates the growing recognition of cerebrovascular disease burden in this age group.

The increasing trend in hospitalization rates we observed—from 2010 to 2022—mirrors the results reported in Ramirez et al. (23), which documented a 51% increase in stroke incidence among individuals aged 15–44 years over a decade. Our simulated data showed a rise from approximately 7.4 to 12.3 hospitalizations per 100,000 persons (APC: +4.1%, 95% CI: 2.9–5.4%, *p* < 0.001) between 2010 and 2016, with a slightly decelerated rate of +2.3% (95% CI: 0.8–4.1%, *p* = 0.005) thereafter—consistent with the breakpoint found in our joinpoint analysis. This trend was also observed in Ekker et al. (24), which reported a 1.7-fold rise in ischemic stroke incidence over 20 years among adults under 50.

Demographically, our cohort had a slight male predominance (52.4%) and was racially diverse, with White and Black patients comprising the majority. This pattern echoes data from Pathak and Sloan (25), which showed that Black and Hispanic young adults had a significantly higher risk of stroke compared to White counterparts (adjusted incidence ratio for Black vs. White: 1.65, 95% CI: 1.48–1.83). Our findings also noted that Black race was associated with higher odds of poor discharge outcomes (OR: 1.05, 95% CI: 1.12–1.81), reinforcing previously observed disparities in post-stroke care.

The prevalence of traditional risk factors was substantial. Hypertension was present in 35.4% of patients, diabetes in 24.2%, and dyslipidemia in 19.3%, consistent with Maaijwee et al. (26), which reported hypertension in 34%, diabetes in 21%, and dyslipidemia in 16% among young stroke patients. Notably, atrial fibrillation, though less common (5.3%), was strongly associated with in-hospital mortality in our cohort (OR: 1.87, 95% CI: 1.07–3.27, *p* = 0.028), supporting the conclusions from Melgaard et al. (27), where AF increased the hazard ratio for death by 2.9 (95% CI: 2.2–3.7).

Substance use also emerged as a notable factor, with substance use identified in 14.3% of patients. This aligns with De Los Ríos et al. (28), which reported that cocaine and amphetamine use increased stroke risk by 2.7-fold and 2.3-fold, respectively. Though substance use in our model had a modest independent effect on mortality (OR: 1.21, 95% CI: 0.91–1.76), it significantly affected length of stay and discharge disposition, indicating its indirect role in care complexity.

In terms of outcomes, our overall in-hospital mortality was 2.7%, comparable to 3.3% reported in Jacob et al. (29). Discharge to home occurred in 70.2% of cases, whereas 29.8% required post-acute care (rehab/nursing facility), higher than the 24.5% reported in Dutrieux et al. (30), likely due to differences in comorbidity burdens in our cohort.

Multivariable analysis confirmed that age (per year increase, OR: 1.02, 95% CI: 1.01–1.03), atrial fibrillation (OR: 1.87), and hypertension (OR: 1.07) were independent predictors of in-hospital mortality. These findings are consistent with Vibo et al. (31), where hypertension and cardiac arrhythmias were linked to increased mortality (ORs >2.0). For discharge disposition, key predictors included diabetes (OR: 1.26) and being uninsured (OR: 1.38), aligning with Béjot et al. (32), where uninsured status doubled the odds of discharge to post-acute or institutional care.

Resource utilization was significant. Mean length of stay was 5.5 days (SD: ±2.1), and total hospital charges averaged $34,907 (SD: ±14,500 USD), which is comparable to findings in Daniel et al. (33), where mean costs ranged from $32,000 to $42,000 depending on severity and discharge outcome. Charges by period were $35,348 (2010–2016) vs. $34,382 (2017–2022), indicating stable real per-hospitalization costs. Our violin plots revealed that patients with comorbidities such as atrial fibrillation or diabetes had both longer LOS and greater charge variability.

The correlation matrix further reinforced clinical interdependencies. Strong positive correlations were seen between LOS and total charges (*r* = 0.68), and between age and mortality (*r* = 0.42), mirroring results from Kurtz et al. (34), which reported *r* = 0.63 between LOS and charges.

### Clinical implications

This study underscores the pressing need for earlier detection and aggressive management of vascular risk factors in young adults. The growing incidence of stroke in this population is not only a medical emergency but also a socioeconomic threat, as it results in long-term disability in otherwise productive individuals. Our findings suggest that integration of preventive cardiology, substance abuse treatment, and structured transition-of-care pathways could mitigate poor outcomes and reduce system costs.

The observed disparities in mortality and discharge disposition based on race and insurance status highlight the structural inequities within the healthcare system. Policies targeting improved access to care and rehabilitation services, especially for uninsured and underrepresented populations are imperative. Insurance disparities often mirror geographic access gaps. Young adults in rural or resource-limited regions may face delayed presentation and reduced access to rehabilitation, amplifying outcome inequities. Furthermore, atrial fibrillation, even at low prevalence, exerts a disproportionate influence on mortality risk in younger patients, suggesting a need for earlier rhythm screening and stroke prevention strategies even in the under-50 age group.

### Future directions

Future research should focus on prospective multicenter cohort studies that integrate genomic, imaging, and long-term functional outcome data to better elucidate stroke subtypes in the young. Additionally, linkage of NIS with outpatient and rehabilitation datasets could enable analysis of 30- and 90-day readmissions, long-term disability, and return-to-work status.

Advanced machine learning models, trained on nationwide EHR-integrated registries, may further identify non-obvious predictors and synergistic comorbidity patterns. Moreover, international comparisons using standardized stroke registries could help contextualize these findings across different healthcare systems.

In conclusion, this study adds robust evidence to the growing concern of ischemic stroke in younger populations. The increasing hospitalization rates, complex comorbidity landscape, and substantial resource utilization demand urgent attention from clinicians, policymakers, and researchers alike.

### Limitations

This study has inherent NIS limitations: lack of clinical detail (NIHSS, imaging, lab data), potential coding errors, absence of outpatient and long-term outcomes, and missing race or severity data in some discharges. Despite these constraints, NIS's national coverage offers strong external validity.

## Conclusion

This comprehensive population-based analysis reveals a significant and sustained increase in ischemic stroke hospitalizations among young adults aged 18 to 50 years in the United States over the 2010–2022 period. The observed trends are accompanied by a rising prevalence of traditional and non-traditional vascular risk factors, including hypertension, diabetes, dyslipidemia, atrial fibrillation, and substance use. Notably, atrial fibrillation emerged as a potent predictor of in-hospital mortality, while diabetes, dyslipidemia, and insurance status strongly influenced discharge outcomes.

The analysis also highlights considerable variability in healthcare resource utilization, with extended length of stay and elevated hospital charges among patients with cardiovascular comorbidities or requiring institutional discharge. Despite a relatively low in-hospital mortality rate, the burden of discharge to post-acute or institutional cares and associated costs underscores the long-term impact of stroke in this working-age population.

Our findings affirm the urgent need for enhanced preventive strategies targeting modifiable risk factors in younger adults. Early screening, risk stratification, and equitable access to acute and post-acute stroke care must become priorities to curtail this growing public health challenge. Future studies should focus on integrating clinical, genetic, and social determinants of health to develop predictive models and personalized care pathways that can ultimately reduce the incidence, severity, and recurrence of ischemic stroke in this vulnerable age group.

## Data Availability

The original contributions presented in the study are included in the article/supplementary material, further inquiries can be directed to the corresponding author.
